# Cost-utility analysis of a consensus and evidence-based medication review to optimize and potentially reduce psychotropic drug prescription in institutionalized dementia patients

**DOI:** 10.1186/s12877-021-02287-7

**Published:** 2021-05-22

**Authors:** Mireia Massot Mesquida, Frans Folkvord, Gemma Seda, Francisco Lupiáñez-Villanueva, Pere Torán Monserrat

**Affiliations:** 1 Servei d’Atenció Primària Vallès Occidental, Direcció d’Atenció Primària Metropolitana Nord. Institut Català de la Salut. Sabadell, Barcelona, Spain; 2 Grup de Recerca Multidisciplinar en Salut i Societat (GREMSAS), accredited by AGAUR (2017 SGR 917), Barcelona, Spain; 3 Tilburg School of Humanities and Digital Sciences, Tilburg University, Tilburg, The Netherlands; 4 Open Evidence Research, Universitat Oberta de Catalunya, Barcelona, Spain; 5 Unitat de Suport a la Recerca Metropolitana Nord, Institut Universitari d’Investigació en Atenció Primària Jordi Gol (IDIAP Jordi Gol), Mataró, Barcelona, Spain; 6 Department of Information and Communication Sciences, Universitat Oberta de Catalunya, Barcelona, Spain

**Keywords:** Cost-benefit analysis, Patient-centered medication review, Dementia, Psychotropic drugs, Nursing homes, Institutionalized patients

## Abstract

**Background:**

Growing evidence shows the effects of psychotropic drugs on the evolution of dementia. Until now, only a few studies have evaluated the cost-effectiveness of psychotropic drugs in institutionalized dementia patients. This study aims to assess the cost-utility of intervention performed in the metropolitan area of Barcelona (Spain) (MN) based on consensus between specialized caregivers involved in the management of dementia patients for optimizing and potentially reducing the prescription of inappropriate psychotropic drugs in this population. This analysis was conducted using the Monitoring and Assessment Framework for the European Innovation Partnership on Active and Healthy Ageing (MAFEIP) tool.

**Methods:**

The MAFEIP tool builds up from a variety of surrogate endpoints commonly used across different studies in order to estimate health and economic outcomes in terms of incremental changes in quality adjusted life years (QALYs), as well as health and social care utilization. Cost estimates are based on scientific literature and expert opinion; they are direct costs and include medical visits, hospital care, medical tests and exams and drugs administered, among other concepts. The healthcare costs of patients using the intervention were calculated by means of a medication review that compared patients’ drug-related costs before, during and after the use of the intervention conducted in MN between 2012 and 2014. The cost-utility analysis was performed from the perspective of a health care system with a time horizon of 12 months.

**Results:**

The tool calculated the incremental cost-effectiveness ratio (ICER) of the intervention, revealing it to be dominant, or rather, better (more effective) and cheaper than the current (standard) care. The ICER of the intervention was in the lower right quadrant, making it an intervention that is always accepted even with the lowest given Willingness to Pay (WTP) threshold value (€15,000).

**Conclusions:**

The results of this study show that the intervention was dominant, or rather, better (more effective) and cheaper than the current (standard) care. This dominant intervention is therefore recommended to interested investors for systematic application.

## Background

Patients with dementia often present neuropsychiatric symptoms. These are known as behavioral and psychological symptoms of dementia (BPSD) and are a common motive for medical consultations, hospital admissions, and nursing home stays [1]. It is estimated that at least 90% of dementia patients will develop some type of BPSD over a period of 5 years.

Several studies [2, 3] have cautioned about the risks of treating BPSD with psychotropic drugs. Psychotropic drug use has been associated with decreased cognitive capacity, rigidity, and somnolence and may result in complications such as pneumonia [4], increased risk of experiencing dementia (around 50%) [5] and mortality [2, 6] in institutionalized dementia patients. These negative consequences suggest that interventions aimed at reducing the risks associated with these drugs might be necessary.

Psychotropic drugs are not the only cause for concern in institutionalized dementia patients; polypharmacy itself is another important factor that must be taken into account to prevent adverse events and potentially inappropriate prescriptions in this population [7].

Numerous studies have demonstrated the effectiveness of medication reviews conducted either by a pharmacist alone or a multidisciplinary team in reducing inappropriate medication [8–10] and its impact [11, 12], either in terms of cost reduction [13] and increased quality of life [12, 14], although not always with favorable results [15–17].

While several studies have demonstrated the effectiveness of interventions aimed at reducing the use of psychotropic drugs in institutionalized dementia patients [18–21], few have evaluated their cost-effectiveness or the impact of these interventions on patients’ quality of life [22]. Additionally, despite showing a reduction in the consumption of psychotropic drugs, no clear benefit has been proven (or the results are contradictory). One potential explanation for this is that these studies evaluated different types of interventions and, therefore, the results will necessarily differ. Another possibility is that they evaluated the cost-utility of a combination of interventions that, in addition to a medication review, included behavioral support intervention. For example, in a study by Ballard et al. [23], patient-centered intervention resulted in increased quality of life even though there was no significant reduction in the consumption of psychotropic drugs in either the intervention or control group. These outcomes are aligned with a 2017 study by Ballard et al. [24] in which social interaction intervention was reported as the only type of intervention that led to increased quality of life. The cost-analysis of the intervention revealed a significant reduction in social and healthcare costs in the intervention group as compared with the control group.

Richter et al. [25], on the other hand, did not analyze cost-effectiveness in their study because the results of the control group were better than those of the intervention group. Harrison et al. found that greater exposure to psychotropic drugs is linked to lower quality of life [26] in contrast with the findings of Ballard et al. [24], in which the reduced use of psychotropic drugs in dementia patients worsened quality of life. However, in another study, Ballard et al. [27] concluded that the discontinuation of antipsychotics may have little or no effect on overall cognitive function. Discontinuation may make no difference to adverse events or quality of life. In a 2016 study, Ballard et al. [28] used mortality analysis to review psychotropic drugs linked to social intervention and identified a reduction in mortality as compared to receiving no intervention.

This study aims to assess the cost-effectiveness of an intervention based on consensus between the primary care pharmacists, GPs and nursing home physicians and nurses involved in the management of dementia patients for optimizing and potentially reducing the prescription of inappropriate psychotropic drugs in this population. The analysis was conducted using the Monitoring and Assessment Framework for the European Innovation Partnership on Active and Healthy Ageing (MAFEIP) tool [29, 30].

## Methods

### Intervention analyzed

The details of the actual intervention analyzed in this study are described elsewhere [31]. In short, the analyzed intervention was performed in the metropolitan area of Barcelona Catalonia (Spain) (MN) between 2012 and 2014. The patients included were institutionalized dementia patients with pharmacological treatment with one or more psychotropic drugs from the Anatomical Therapeutic Chemical Classification System (ATC) code N of the World Health Organization (WHO) [32] for 3 months or longer. It was a structured, patient-centered medication review by a multidisciplinary team (primary care pharmacists, GPs, nursing home physicians and nurses) aimed at reducing inappropriate psychotropic drug prescriptions based on a therapeutic guideline for treating BPSD (created by a multidisciplinary team) as compared to standard care. Before implementing the medication review, one general practitioner (GP) and one primary care pharmacist underwent a training phase focused on managing patients with BPSD. Then, before each scheduled visit with the nursing home physician and nurse, the medication the patient was receiving was evaluated by the GP and pharmacist to detect any incidents related to the prescription (e.g., duplicates, inappropriate drugs) and to determine which prescription-related aspects required evaluation of the patient for decision-making (continue or discontinue a drug). As for the working session in the nursing home, before conducting the medication review (of all drugs included in the therapeutic plan), the GP and pharmacist met with the nursing home physician and nurse to establish the patient’s current status with regard to his/her prognosis, level of dependence, and frailty. This was done to facilitate decisions on the need to adapt the intensity of treatment, change treatment, or discontinue treatment based on the risk-benefit ratio for the patient. Patient assessment included the severity of dementia (Global Dementia Scale [GDS]), level of dependence (Barthel index score), cognitive state (Pfeiffer test), and prognosis (in end-of-life patients). Treatment assessment included the indication, effectiveness, and safety (duplicates, interactions, contraindications) of the drugs prescribed. Based on these evaluations, changes in the patient’s treatment plan considered to be appropriate and relevant were proposed. Follow-up of the consensus changes was carried out at 1 and 6 months to determine whether any psychotropic drugs that had been discontinued after the interview should be restarted, the dosage of drugs that had been reduced should be increased, or a new psychotropic drug should be initiated. Over the course of the intervention, the mean number of drugs prescribed per patient decreased from 8.04 at baseline to 5.86 at 6 months and from 2.71 to 2.01 for psychotropic drugs.

### Model description

The analysis was conducted using the Monitoring and Assessment Framework for the European Innovation Partnership on Active and Healthy Ageing (MAFEIP) tool. In MAFEIP Users Guide Manual [33] describes the basic principles of the tool to better understand the tool and how it works. In this manual is described the MAFEIP tool is founded on the principles of Decision Analytic Modelling (DAM), an approach commonly used to evaluate the health and economic impact of healthcare innovations. More precisely, MAFEIP is based, by default, on a generic three-state Markov model, which provides the flexibility required to be adaptable to a large number of studies that focus on a variety of objectives, implement different interventions and target different cohorts of individuals with different demographic or disease characteristics.

### Transition states

**Fig. 1:**
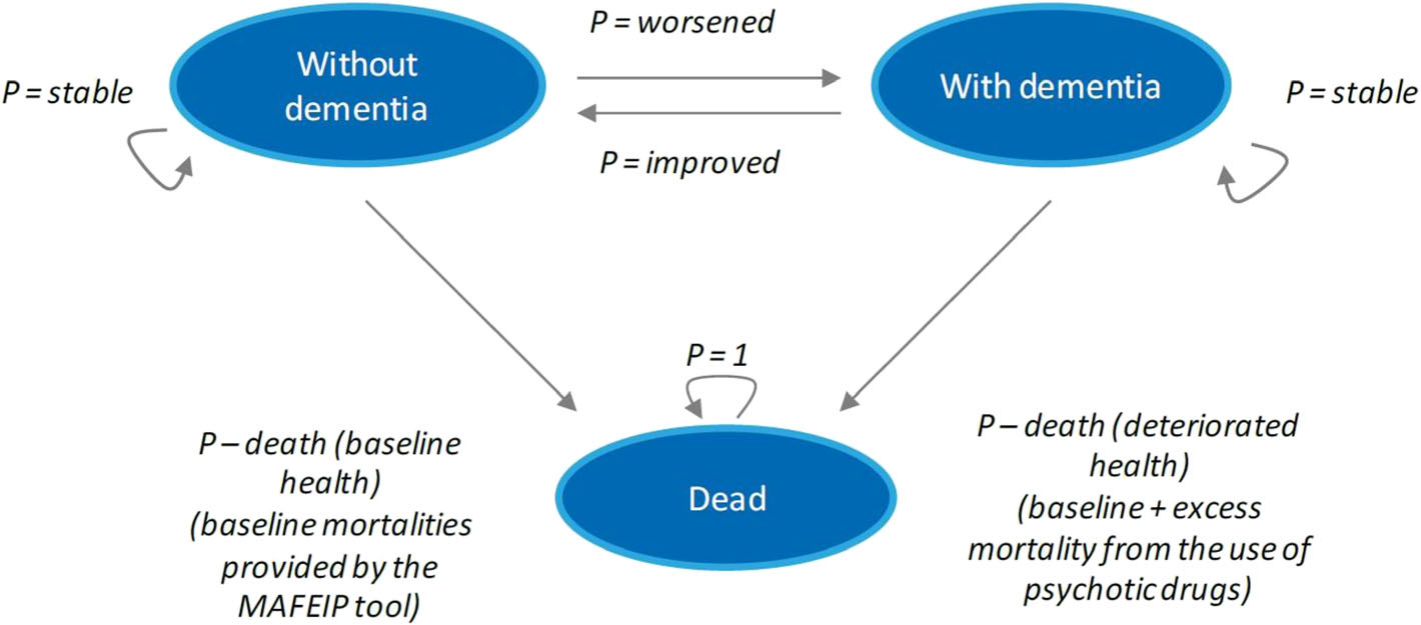
Three-state Markov model used in MAFEIP tool

The outcomes for the intervention and control group are calculated by simulating the health status of the target population as it transitions through the three health states defined in the Markov model: baseline health, deteriorated health, and dead (Fig. 1). Each health state is defined by an amount of resource use and quality of life (utility). This represents the average resource use and quality of life of a patient in that health state.

In the simulation, the population moves through the Markov states based on four transition probabilities: 1) Transition from the baseline health state to the deteriorated health state represents a patient becoming ill (i.e., the incidence of the deteriorated health condition); 2) Patients move from the deteriorated health state to the baseline health state when their illness is cured (i.e., the rate of recovery back to baseline health); 3) Transition to the dead state from the baseline state (i.e., baseline mortality in the target population); 4) Transition to the dead state from the deteriorated health state (i.e., excess mortality in the population with deteriorated health).

The MAFEIP tool builds up from a variety of surrogate endpoints commonly used across different studies in order to estimate health and economic outcomes in terms of incremental changes in quality adjusted life years (QALYs), as well as health and social care utilization.

### Three state Markov model

A highly adaptable Markov model with initially three mutually exclusive health states (baseline health, deteriorated health and death) forms the basis of the tool, which draws from an extensive database of epidemiological, economic and effectiveness data. It also allows for further customization through remote data entry, thus enabling more accurate and context-specific estimation of intervention impact.

In our study, the three-state Markov model of the MAFEIP tool was used to describe patients without dementia (baseline health state), patients with dementia (deteriorated health state), and death. Nonetheless, considering there were no patients without dementia, and the probability that patients with dementia would improve and go to the baseline health state was zero. In the MAFEIP-tool it is not possible to dismiss this state. The study combined data from nursing homes with data collected from external sources to populate the model with values for transition probabilities and relative risk of mortality. Incidence rates were derived from data on the prevalence of dementia in institutionalized patients in our reference area. Moreover, estimates on mortality rates were derived from the findings of the DART-AD [2] clinical trial, which reported higher survival rates in patients who were given placebo drugs instead of neuroleptics.

### Costs and utility values

Cost estimates were based on scientific literature, the eSalud database [34] and expert opinion. The cost estimates are direct costs, including medical visits, hospital care, medical tests and exams and drugs administered, among other concepts.

The healthcare costs of patients using the intervention were calculated by means of a medication review that compared drug-related costs of the patients before, during, and after the use of the intervention.

Intervention cost estimates were based on the amount of time spent by the physician, pharmacist, and/or nurse with the patients.

Regarding utility values, the study did not focus on utility calculations but used secondary sources to provide general and averaged estimates [35]. Utility values of the baseline group describe data collected on health-related quality of life (HR-QoL) [36] for people from the same country and of the same age group who do not have dementia. Since the intervention has been shown to have an impact on mortality rates rather than HR-QoL, utility values for the control group and intervention group are similar. (Utility for control and intervention group: baseline 0.70 and deteriorated 0.27).

The cost-utility analysis was performed from the perspective of a health care system with a time horizon of 12 months. It was focused on calculating the incremental cost-effectiveness ratio (ICER) of the intervention and comparing it to the standard of care. In addition to the analyses on the outcomes; we have included the willingness to pay as a threshold to show the relation between this willingness to pay and the cost-effectiveness of the outcomes.

### Model and base case

As previously mentioned, the use case takes into account the three-state Markov model depicted in Fig. 1.

Each health state describes institutionalized patients between 65 and 90 years of age. The baseline health state describes patients without dementia whereas the deteriorated health state describes patients with dementia. Records show that the prevalence of dementia in institutionalized patients in our reference area is 40%. This is adopted as the incidence rate for both the preintervention group and postintervention group since the intervention does not focus on reducing the rate of incidence of dementia and therefore does not affect it. In this use case, it was assumed that patients cannot completely recover once they already have dementia and, thus, recovery rates were set to 0 for both groups. (Transition probabilities: intervention and control group, incidence rate: 40 and recovery rate: 0).

Mortality rate estimates were derived from the findings of the DART-AD [2] clinical trial, which reported higher survival rates in patients who were given placebo drugs instead of neuroleptics. Based on the clinical trial, the cumulative probability of survival at 12 months was 70% in the preinetrvention group (those who continued treatment with neuroleptics) vs. 77% in the postintervention group (those who withdrew neuroleptics). Moreover, the results show that the difference between the groups was greater after longer periods: at 24 months, survival rate was 46% vs. 71% and at 36 months, it was 30% vs. 59%. The mortality rates were obtained by first calculating the mean of all the survival rates given (49% vs. 69% or 0.49 vs. 0.69) and then subtracting the mean survival rates from 1. (Calculated mortality rate: 0.51 vs. 0.31).

### Relative risk of mortality

The MAFEIP tool uses another measure for estimating mortality in a given population: the relative risk of mortality (RR). This is calculated by dividing the mortality rates in the use case by the mortality for a reference condition. In this particular use case, the reference condition is the age-dependent, all-cause mortality of the Spanish population as recorded in the Human Mortality Database (https://www.mortality.org/). The RR values were a baseline health of 1 and deteriorated health of 1.31 for the postintervention group, and 1 and 1.51 for the preintervention group, respectively.

Patients in the baseline health state had an RR of 1. This is the default value and it prompts the tool to simply use data that is stored in the Human Mortality Database. Those in the deteriorated health state, however, were given an RR greater than 1. Choosing the value RR > 1 leads the tool to interpret this as excess mortality to be added to the existing data from the Human Mortality Database (i.e., mortality rate from database + excess mortality from use of neuroleptics).

Across all patient groups, those who had dementia (deteriorated state) and were given neuroleptics (preinetrvention group) had the highest mortality rate (1.51 is higher than baseline and higher than the postintervention group). Mean patient age was 87.09 years (SD: 6.795), and 75% (*n* = 180) were women. There were no significant differences in patient age between the participating nursing homes.

### Computing the costs

The drug-costs were calculated considering the drug prescribed, dose and frequency for then calculate the number of boxes according to the length of treatment or if the length was more than a year it was considered a maximum length of 1 year period time. This was calculated for each drug from patient pharmacotherapy. The final cost was the sum of the number of boxes multiplied by its cost.

Intervention cost estimates were based on the amount of time spent by the physician, pharmacist, and/or nurse with the patients. The total sum of intervention costs calculated from the staff spending 30 min with each patient, in accordance with the therapeutic plan, came to €11,654.40 or €48.56 per patient. Since the patient does not have to pay for the therapeutic guide itself before it is used, the intervention one-off costs were left at 0.

Cost estimates described the average healthcare costs per dementia patient per year as ranging from approximately €3596.88 to €5130.90, adapted from social-economic studies of Alzheimer disease and vascular dementia patients based in Spain [35, 37].

However, for this use case, the intervention specifically aimed to reduce the amount of neuroleptic drugs given to patients. A study of the costs of this intervention included a medication review that compared drug-related costs of the patients before, during, and after the use of the intervention. For this reason, only drug-related costs were considered in the MAFEIP tool.

Drug-related costs per patient were calculated to be €2265.68 before the intervention, €1720.77 at 1 month after intervention and €1539.90 at 6 months after the intervention. All other costs were considered constants. The Table 1 summarizes the results of the medication review and the rest of model parameters. These are transferred to the MAFEIP tool as follows:

**Table 1:** Summary table of model parameters included

Demographic characteristics of target group	Mean age	87.09 (SD 6.675)
	Sex	75% women
Geographic characteristics of target group	Country	Spain
	Region	Catalonia
Target population minimum age	65	
Target population maximum age	90	
	**Before Intervention**	**After intervention**
Incidence rate of dementia in institutionalized patients in our area	40%	40%
Recovery rate	0%^a^	0%^a^
Relative Risk of Mortality in baseline health	1	1
Relative Risk of Mortality in dementia patients^b^	1.51	1.31
Intervention one-off costs (per patient)		0
Intervention recurrent costs (per patient and year)		48.56€
Healthcare costs baseline health	0	0
Healthcare costs dementia patients	2266 €	1630 €
Utility	0.70	0.27

^a^Recovery rate was considered 0% because once a patient is diagnosed with dementia there is no recovery. ^b^Estimates on mortality rates were derived from the findings of the DART-AD [2] clinical trial. Baseline health costs were left at 0 since the intervention only focuses on patients that are already in a deteriorated health state (they already have dementia). The healthcare cost deteriorated health after intervention were calculated as the drug-related costs mean per patient recorded at 1 and 6 months after the intervention

## Results

**Fig. 2:**
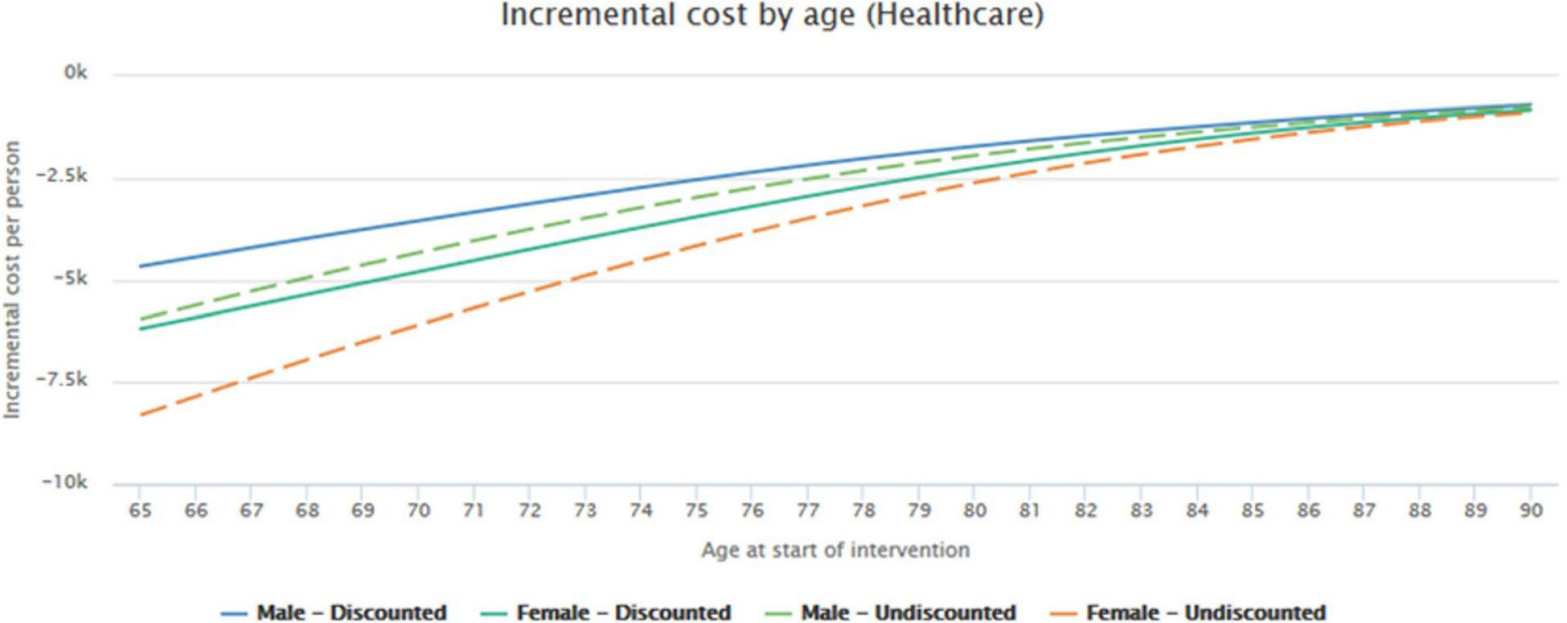
Incremental cost by age

We will present the results by showing the incremental costs planes, which show the difference between the cost that a person with a specific age and gender would have if this person received the intervention minus the cost that would have occurred if this person followed current care. In addition, the incremental effects values show how much quality of life is gained when the intervention is used instead of standard care, comparing them by age-gender group. Next, the ICER will be presented, which is the incremental cost-effectiveness ratio. This is presented as a blue dot and shows whether the intervention is not acceptable (more costly and/or less effective) or acceptable (less costly and/or more effective). The incremental costs by age all fall below zero (negative), as shown in Fig. 2. Negative incremental cost can be interpreted as “savings” that the intervention may generate, whereas positive results can be interpreted as additional costs required by the intervention as compared to standard care. The figure below shows that the incremental costs by age are all negative, which implies that the intervention (having a therapeutic guideline and plan) is cheaper than standard care (continuing with neuroleptic prescriptions only). The graph below also shows that the costs remain negative across the age range (65 to 90 years), although they do increase as age increases (depicted by upward curve).

**Fig. 3:**
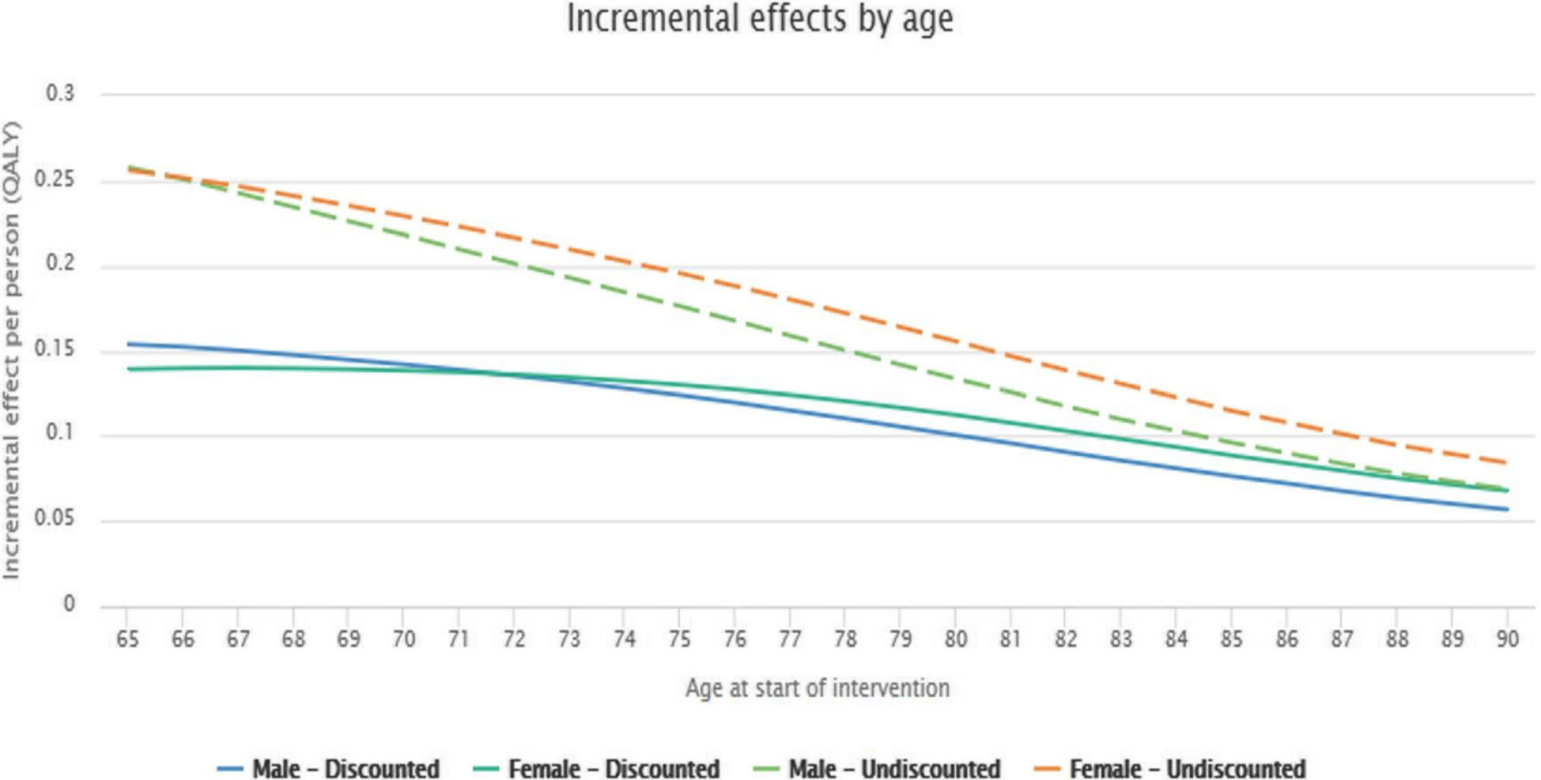
Incremental effects by age

The graph portraying incremental effects by age (Fig. 3) shows how many QALYs each age group in the target group was able to benefit from if the intervention was used. Since this use case did not focus on impact on QALYs, the values in the graph remain close to the earlier input value (0.7 baseline and 0.27 deteriorated state). The graph also illustrates that QALYs are lower at an older age.

**Fig. 4:**
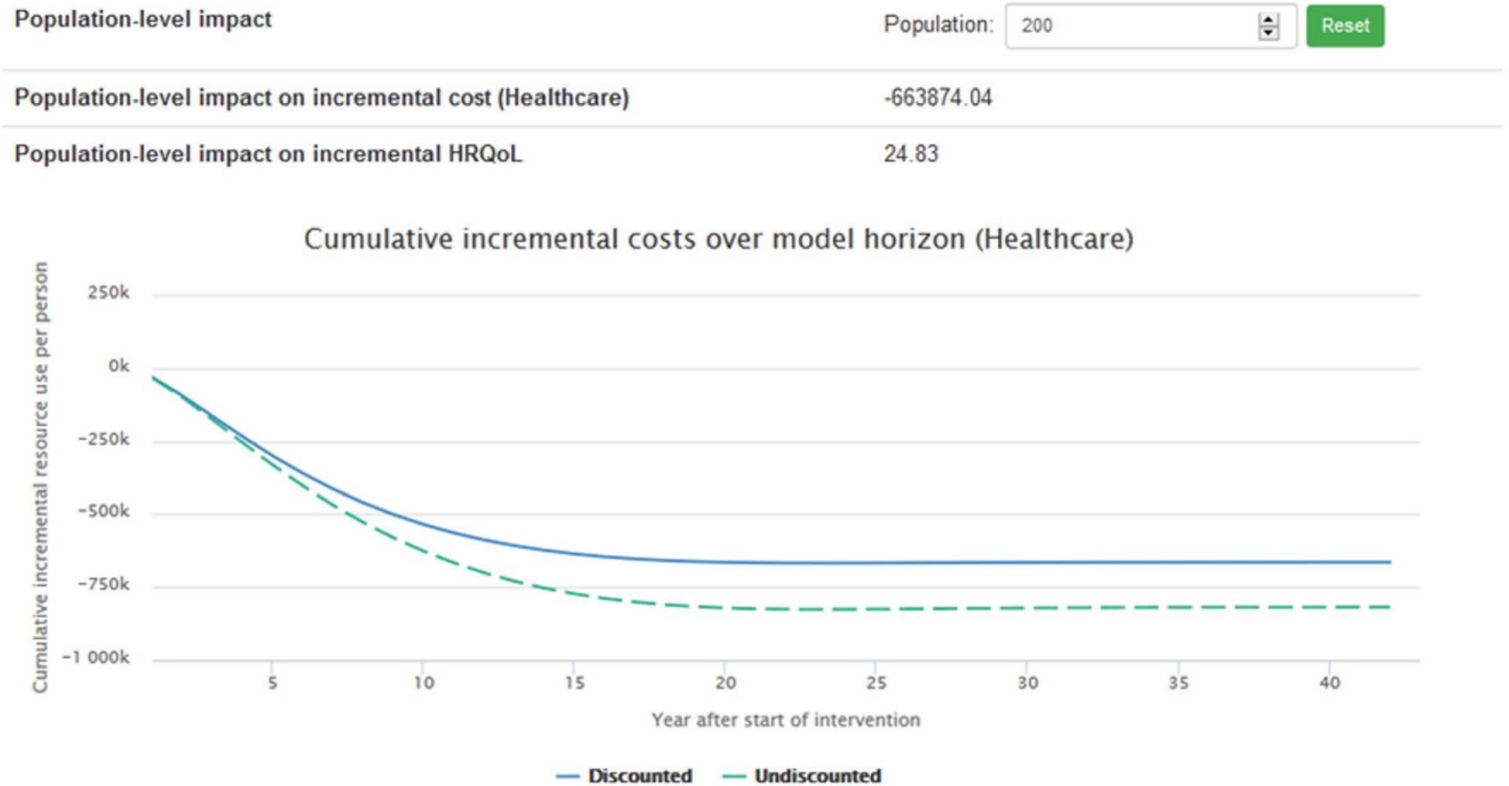
Cumulative incremental cost

**Fig. 5:**
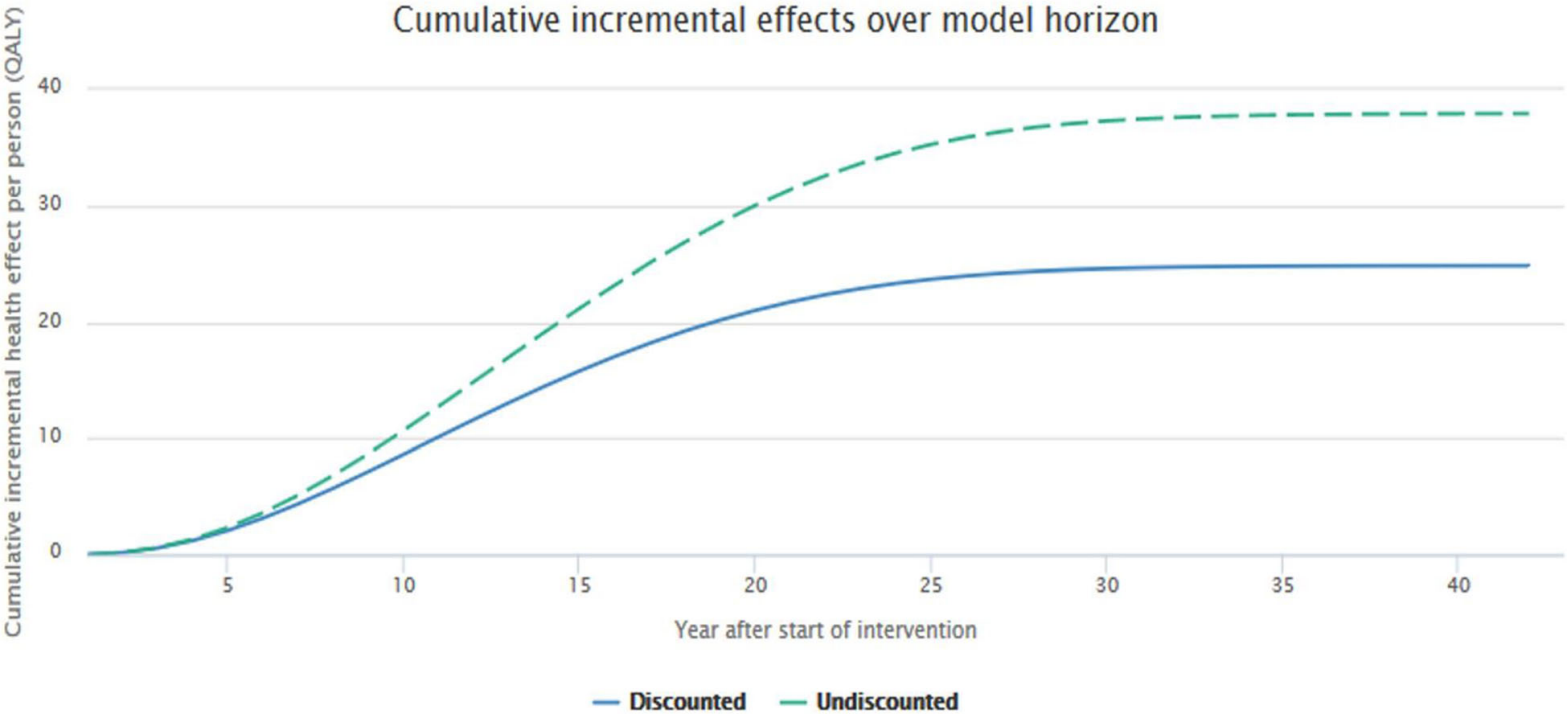
Cumulative incremental effects

Figures 4 and 5 presents an extension of the previous results by showing the population-level impact on costs and effects over a period of time (40 years). A sample population of 200 was chosen.

**Fig. 6:**
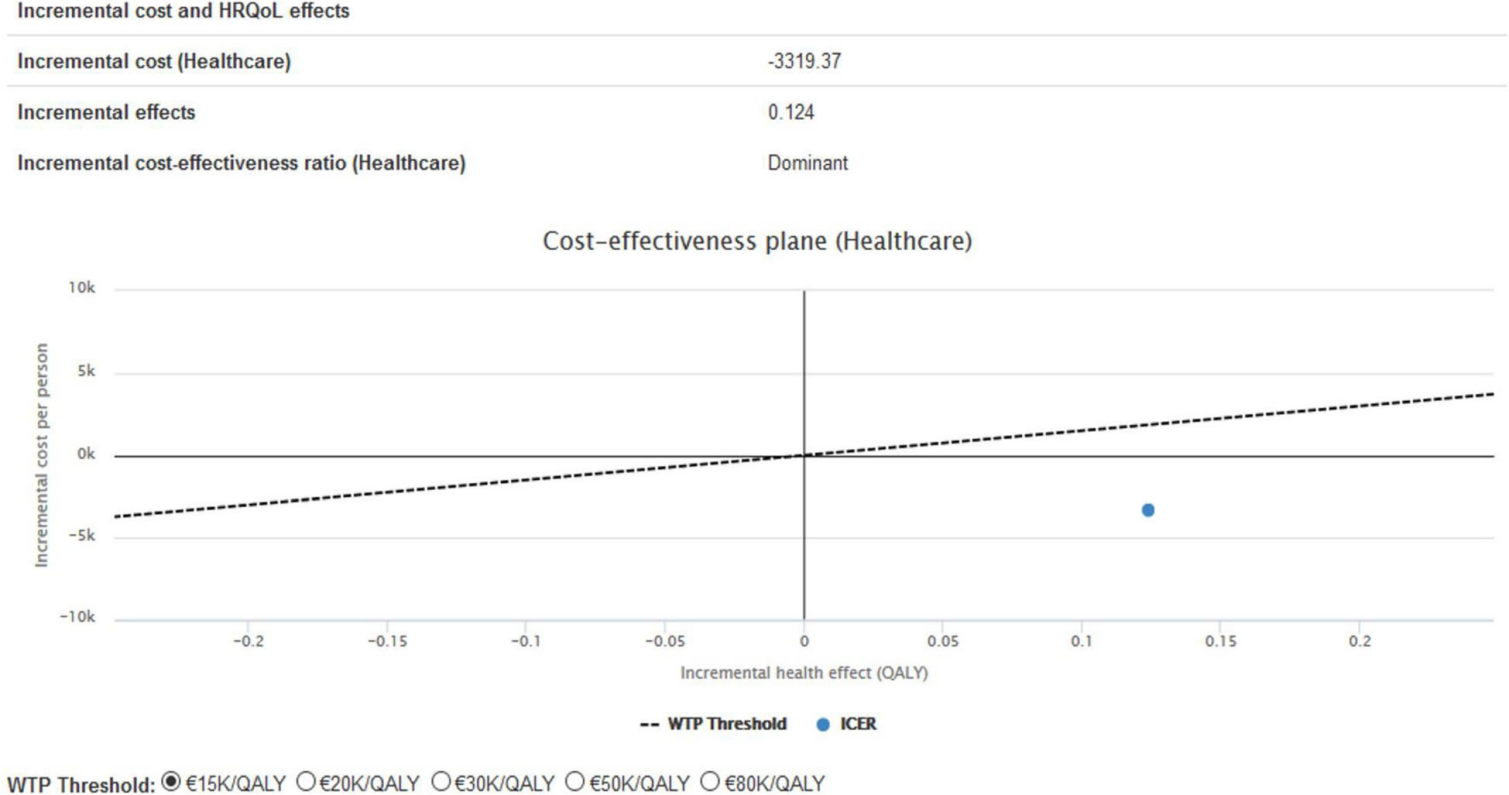
Cost-effectiveness plane

The ICER takes into account both the potential incremental costs and incremental effects that intervention may provide. In this example, the ICER is described as “dominant” and its exact value (represented by the blue dot in Fig. 6) is located in the lower-right quadrant of the graph. If an intervention is “dominant” it means that it is better (more effective) and cheaper than the current (standard) care. It also means that the intervention is (always) accepted and, therefore, possibly of value to interested investors.

Moreover, the WTP threshold describes the maximum amount patients are willing to pay given a certain QALY. In our study we have used 15 k/QALY as he WTP threshold. When the ICER is less than the WTP, it means that the intervention is cheaper than what patients are willing to pay for it (also making the intervention accepted).

**Fig. 7:**
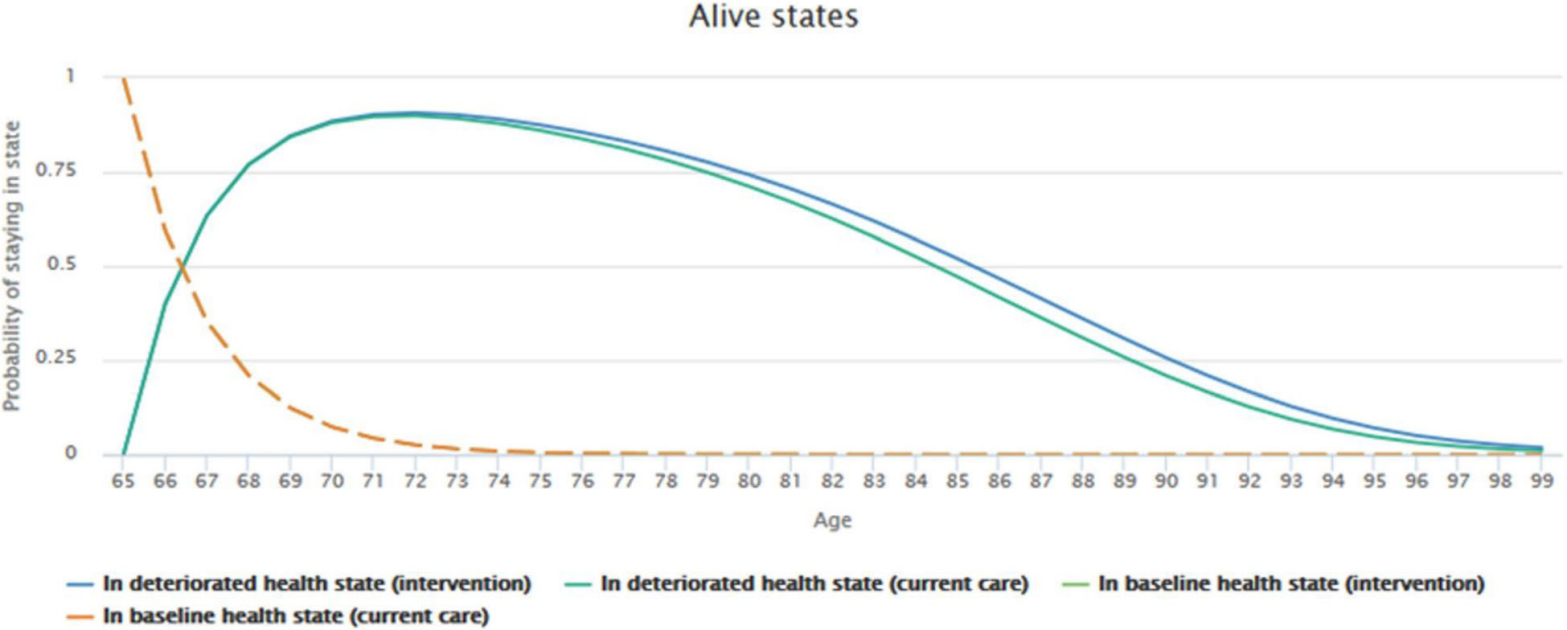
Patient flow through model states (Alive states)

**Fig. 8:**
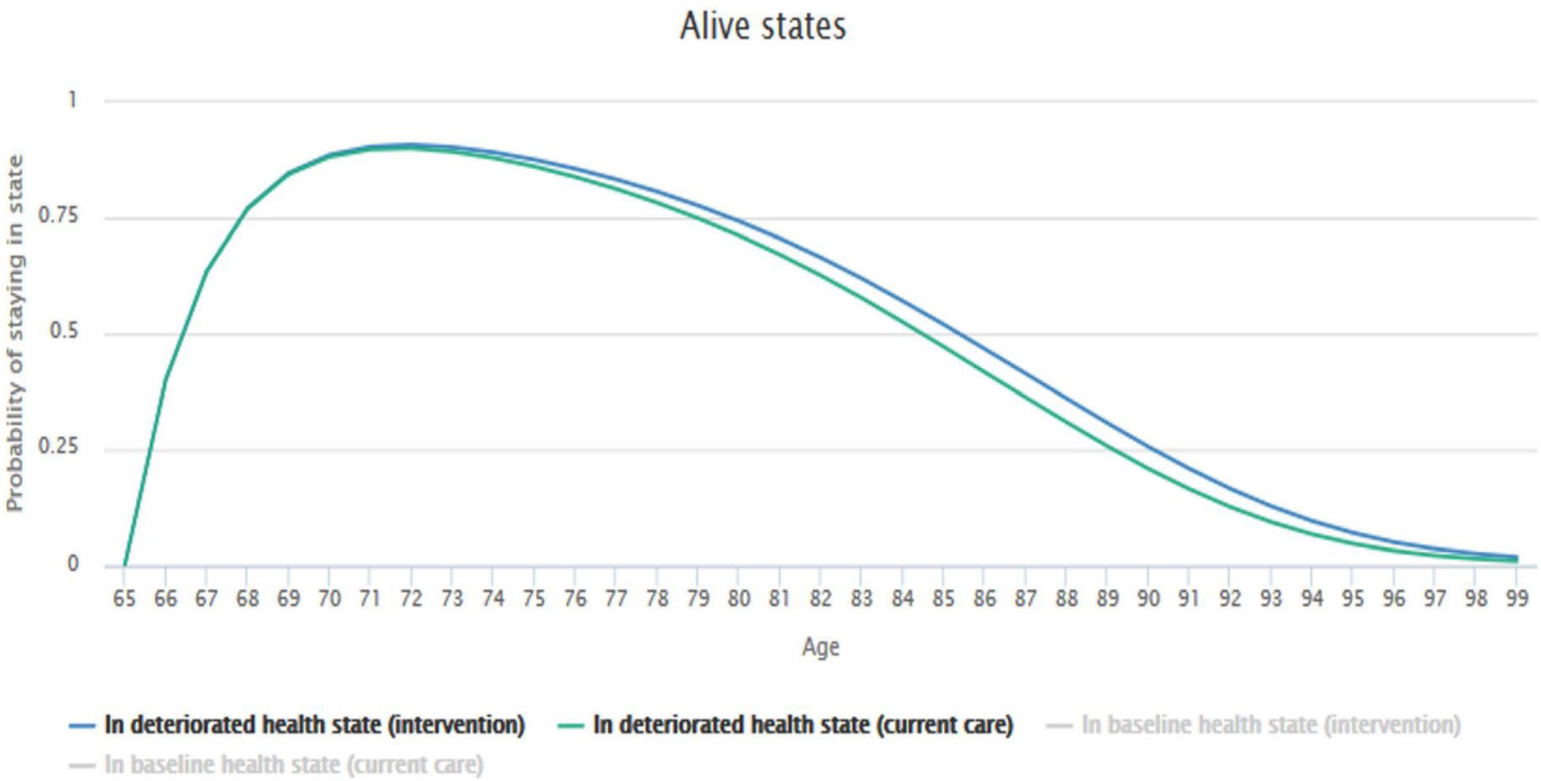
Patient flow through model states (Alive states)

Figures 7 and 8 reflect the simulation results based on the transition probabilities described earlier. The graphs illustrate how likely it is that a patient from a given age group and health state will remain in an alive state or move to the dead state. Mortality rates of patients without dementia (baseline health state) were set to the default value of 1. The tool then took age- and country-dependent, all-cause mortality rates from the Human Mortality Database.

In order to better compare the effect of the intervention (therapeutic plan) vs. current care (neuroleptics) specifically for patients in the deteriorated state (with dementia), the plots depicting the baseline health states were deselected (Fig. 8). The graph shows that there are better chances of remaining in an alive state when using the intervention rather than current care.

**Fig. 9:**
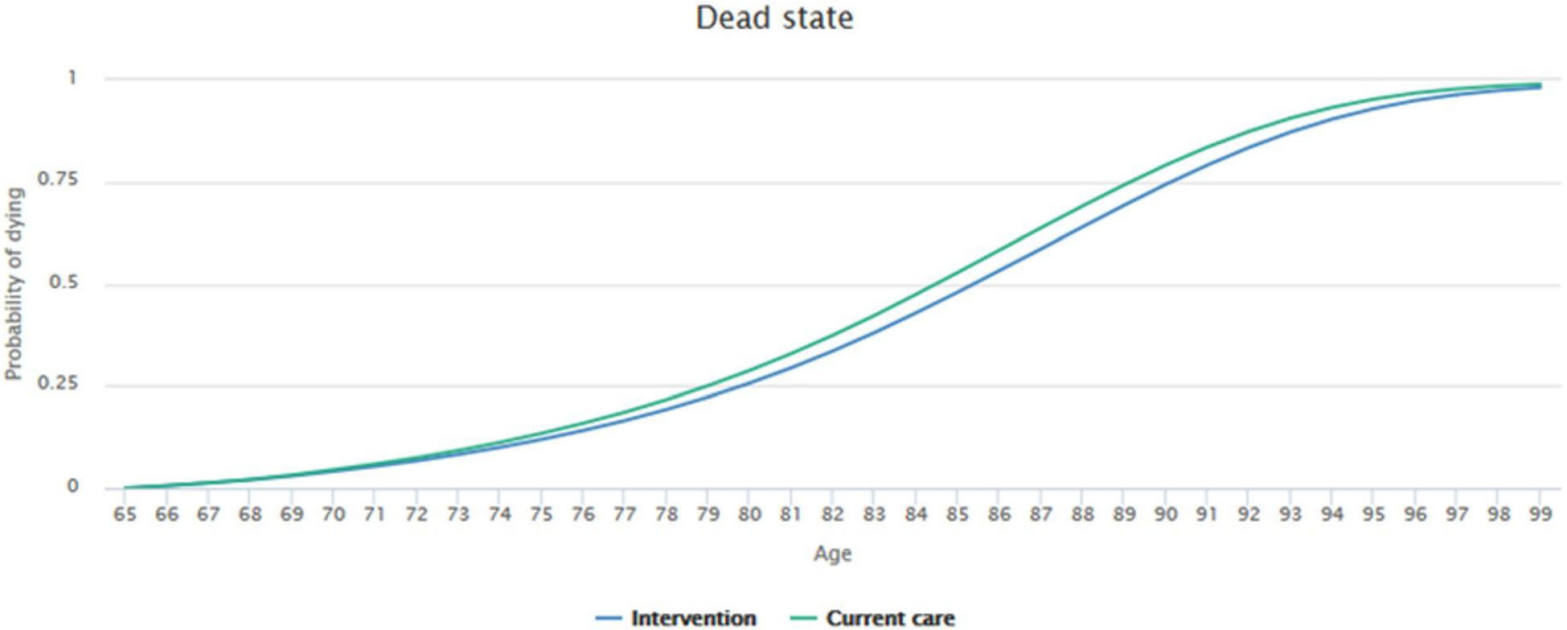
Patient flow through model states (Dead state)

Figure 9 describes the probability of patients moving to the dead state (by age group). The graph illustrates how patients using the intervention have lower chances of dying when compared to patients still being given neuroleptics (current care).

## Discussion

This study aims to assess the cost-effectiveness of an intervention based on consensus between the primary care pharmacists, GPs and nursing home physicians and nurses involved in the management of dementia patients for optimizing and potentially reducing the prescription of inappropriate psychotropic drugs in this population. Intervention based on specific, evidence-based therapeutic guidelines designed by a multidisciplinary team and implemented through consensus together with a patient-centered clinical medication review has proven to be a dominant or cost-effective intervention. This means that the intervention is better (more effective) and cheaper than the current (standard) care. The results describe an intervention that is always recommended to be accepted, even with the lowest given WTP threshold value (€15,000).

The positive results obtained in this cost-utility analysis are due to the number of drugs and psychotropic drugs deprescribed and, based on the evidence [2], their impact on mortality outcomes in institutionalized dementia patients. The mean number of drugs prescribed per patient decreased from 8.04 at baseline to 5.86 at 6 months and from 2.71 to 2.01 for psychotropic drugs.

This reduction could be due to the methodology used: patient-centered clinical medication review by a multidisciplinary team, decision-making based on a single guide used by all team members, and the solving of different issues through an on-site meeting with all the managers responsible for patient care, in which the therapeutic objectives were shared, thus favoring joint decision making. A systematic review by Kwak et al. [17] that analyzed all the economic studies on medication review interventions conducted by pharmacists in nursing homes did not obtain a clear conclusion due to the heterogeneity of the studies included. In the discussion, however, it was pointed out that studies with positive economic results were based on a collaborative model, in which all the team members had a defined role, the objectives and guides were shared and an effective communication method was employed.

Specifically, as regards the reduction of psychotropic drugs, it should be noted that at the beginning of the study we verified the prevalence of psychotropic prescriptions and polypharmacy in dementia patients and that it was higher than that described by other authors [38–40]. For this reason, the intervention is probably cost-effective at the time of implementation and after 6 months. The impact of the intervention and its cost-utility after a longer period and when the prevalence of drugs is lower still must be studied.

This fact might be reflected in the Ballard et al. study [24] in which discontinuance of psychotropic drugs in the study population had a negative impact on patients’ quality of life. In the discussion, the authors note that the results must be due to a general reduction in the prescription of psychotropic drugs over the previous 5 years, leading to a low prevalence (18%) of psychotropic drugs among the study population. This shows that patients that currently have a psychotropic drug prescribed probably have more severe neuropsychiatric symptoms than in the past.

The results obtained from this cost-utility study cannot be compared to the results of other cost-effectiveness studies due to the different methodologies used and interventions performed [23, 28, 41, 42], as well as the type of patients included [23] and results analyzed.

As for its limitations of this analysis is the very nature of the study since it is not a clinical trial but a pre/post intervention study. Morover, as a limitation, the progress of patients condition has not been taken into account, in the original article describing the intervention no further clinical evaluations were carried out after the initial patient assessment; but it shows that deprescription decisions taken during the intervention remained in the sample over some months, Another limitation is that the model we use does not match perfectly the baseline health state of CUA model, because there were no patients without dementia, and the probability that patients with dementia would improve and go to the baseline health state was zero. Lastly, common to all cost-utility analyses, is that societal benefits and costs are not taken into account,. Despite these limitations, as for its strengths, this study makes it possible to compare different ICERs of the health interventions and policies.

## Conclusion

The results of this analysis showed that our intervention was dominant, that is, better (more effective) and cheaper than the current (standard) care. This also means that the intervention is always better than the standard care and, therefore, possibly of value to interested decision makers since the prevalence of dementia is on the rise due to an ever-larger aging population. Having demonstrated its cost-effectiveness, the implementation of a guideline for reducing prescriptions based on the consensus of all the professionals involved in patients’ care leads to proven benefits for both the patients themselves and the healthcare system.

## Data Availability

The datasets generated and/or analyzed in this study are not publicly available due to the policies of our institution but are available upon request from the corresponding author.

## Abbreviations

ATC: Anatomical and therapeutic chemical classification system
BPSD: Behavioral and psychological symptoms of dementia
DAM: Decision analytic modeling
GDS: Global dementia scale
GP: General practitioner
HR-QoL: Health-related Quality of life
ICER: Incremental cost-effectiveness ratio
MAFEIP: Monitoring and Assessment Framework for the European Innovation Partnership on Active and Healthy Ageing tool
QALYS: Quality adjusted life years
RR: Relative risk
WHO: World Health Organization
WTP: Willingness to pay
